# Novel heteroduplex method using small cytology specimens with a remarkably high success rate for analysing EGFR gene mutations with a significant correlation to gefitinib efficacy in non-small-cell lung cancer

**DOI:** 10.1038/sj.bjc.6603396

**Published:** 2006-10-17

**Authors:** F Oshita, S Matsukuma, M Yoshihara, Y Sakuma, N Ohgane, Y Kameda, H Saito, K Yamada, E Tsuchiya, Y Miyagi

**Affiliations:** 1 Department of Thoracic Oncology, Kanagawa Cancer Center, Nakao 1-1-2, Asahi-ku, Yokohama 241-0815, Japan; 2 Molecular Pathology and Genetics Division, Kanagawa Cancer Center, Nakao 1-1-2, Asahi-ku, Yokohama 241-0815, Japan; 3 Department of Pathology, Kanagawa Cancer Center, Nakao 1-1-2, Asahi-ku, Yokohama 241-0815, Japan; 4 Laboratory for Molecular Diagnostics, Kanagawa Cancer Center, Nakao 1-1-2, Asahi-ku, Yokohama 241-0815, Japan

**Keywords:** EGFR, mutation, cytology, lung cancer, gefitinib

## Abstract

We conducted a feasibility study to examine whether small numbers of cancer cells could be utilised for analysis of the EGFR gene status using the loop-hybrid mobility shift assay, which is a modified heteroduplex technique. Cytology specimens obtained by transbronchial abrasion were successfully used for analysis of the EGFR gene status in 50 of 52 (96.2%) patients diagnosed with class V non-small-cell carcinoma. Furthermore, the relationship between the EGFR gene status and clinical outcome was analysed in 25 patients treated with gefitinib. Overall, 10 of 11 patients with EGFR mutations in exon 19 or 21 showed tumour regression with gefitinib treatment, compared to only two of 14 patients with wild-type EGFR. The response rate was significantly higher in the EGFR mutation group than in the wild-type EGFR group (90.9 *vs* 14.3%, *P*=0.00014). Logistic regression analysis revealed that EGFR mutations in cytology specimens represented an independent predictor of the gefitinib response. The overall and progression-free survivals were significantly longer in the EGFR mutation group than in the wild-type EGFR group (*P*<0.05). In conclusion, cytology specimens could be useful for analysing the EGFR status in the majority of patients with non-small-cell lung cancer to determine whether they are likely to benefit from gefitinib treatment.

Non-small-cell lung cancer (NSCLC) is the leading cause of cancer deaths in Japan. Current chemotherapy regimens for metastatic NSCLC are not particularly effective, and the disease cannot be cured even with the most effective platinum and new combination chemotherapies. Recent progress in lung cancer biology has led to the development of small-molecule inhibitors of target proteins involved in proliferation, apoptosis and angiogenesis. The epidermal growth factor receptor (EGFR) superfamily was identified early on as a potential therapeutic target in solid tumours. Given the biological importance of the EGFR molecular network in carcinomas, several molecules that can inhibit the EGFR tyrosine kinase domain have been synthesised. These inhibitors include gefitinib and erlotinib, both of which are orally active and can produce an objective response in previously treated or untreated advanced NSCLC (Fukuoka *et al*, 2003; Kris *et al*, 2003; Miller *et al*, 2004; Perez-Soler *et al*, 2004). A previous randomised study demonstrated that addition of gefitinib to standard platinum-based chemotherapy did not improve the outcome of patients with NSCLC (Giaccone *et al*, 2004; Herbst *et al*, 2004). Furthermore, consolidation with gefitinib did not improve the outcome in NSCLC patients receiving full-dose chemotherapy and thoracic radiotherapy followed by docetaxel (Kelly *et al*, 2005). A placebo-controlled study also failed to demonstrate that gefitinib improved survival (Thatcher *et al*, 2005). On the basis of these results, the use of gefitinib has not been recommended for treatment of patients with NSCLC in Europe and the United States.

Meanwhile, responders to chemotherapy generally have a better prognosis than non-responders, and chemosensitivity is an important factor in deciding which patients should receive chemotherapy. Responsiveness to gefitinib is a characteristic of distinct subgroups of patients, such as women, patients who have never smoked, patients with adenocarcinoma and Asians (Kris *et al*, 2003; Miller *et al*, 2004; Thatcher *et al*, 2005). Although the level of EGFR protein expression is not associated with the gefitinib response, specific missense and deletion mutations in the tyrosine kinase domain of the EGFR gene have been reported to be associated with gefitinib sensitivity (Lynch *et al*, 2004; Paez *et al*, 2004). A retrospective study demonstrated that NSCLC patients with EGFR mutations have a better outcome with gefitinib treatment than patients with the wild-type EGFR gene (Mitsudomi *et al*, 2005). The National Cancer Institute of Canada Clinical Trial Group BR.21 placebo-controlled study demonstrated a survival advantage for patients with NSCLC who received erlotinib after other treatments had failed (Shepherd *et al*, 2005). That study also evaluated the EGFR gene status and analysed its relationship with the clinical outcome. It was concluded that the presence of an EGFR mutation is not indicative of a survival benefit, but may increase the responsiveness of patients with NSCLC to erlotinib treatment (Tsao *et al*, 2005). Therefore, it seems important to limit gefitinib or erlotinib treatment to NSCLC patients with EGFR mutations.

Although tumour tissue, such as that obtained by surgical resection or transbronchial biopsy, has usually been used for analysis of EGFR mutations in lung cancer, a small number of patients with NSCLC can be diagnosed purely on the basis of cytology using small numbers of cancer cells. In a previous large-scale study examining the benefits of erlotinib treatment for NSCLC, analysis of EGFR mutations using cytology specimens was possible in only 197 of 731 (27%) patients (Tsao *et al*, 2005). In addition, in a study that demonstrated a 30% objective response to gefitinib as a first-line treatment for NSCLC, the EGFR gene status could be examined using cytology specimens in only 13 of 40 (32.5%) patients (Niho *et al*, 2006). These low numbers may reflect the difficulties associated with obtaining biopsy specimens by bronchoscopic examination for the diagnosis of NSCLC. Thus, in order to apply the EGFR mutation strategy to all patients, a new method that requires only a small number of cells is necessary. A novel method for the detection of small deletions as well as point mutations in DNA fragments based on retarded migration of loop hybrid (LH) DNA has recently been developed (Matsukuma *et al*, 2006). The LH DNA is formed by hybridisation of a single-stranded DNA fragment to a complementary strand with the deletion of seven nucleotides. In comparison with the normal duplex DNA, the LH DNA shows strikingly retarded electrophoretic migration in a native polyacrylamide gel owing to the presence of a single-stranded nucleotide loop situated in the middle of the duplex. The nucleotide sequence of the loop affects the mobility of the LH DNA to such an extent that displacement of the loop position by a single nucleotide is distinguishable. These anomalous electrophoretic properties of LH DNA have been adapted for the detection of hotspot point mutations of the EGFR gene in lung adenocarcinoma. The new mutation detection system, known as the LH-mobility shift assay (LH-MSA), is very sensitive and may be useful for molecular diagnosis of clinical cancer specimens. Thus, in order to develop a method for analysing the EGFR gene status in large numbers of patients and applying the results to decide whether gefitinib treatment is indicated, we performed a feasibility study to clarify whether the LH-MSA using small numbers of cancer cells could be applied for analysis of EGFR mutations, and then further evaluated its prediction ability of the EGFR gene mutation status relative to the gefitinib response.

## PATIENTS AND METHODS

This study was approved by the Institutional Review Board of Kanagawa Cancer Center.

### Patients

A total of 52 patients with cytologically proven class V NSCLC were entered into the present study. Written informed consent for EGFR genetic analysis of the tumour tissue or cancer cells was obtained from each patient. Some patients received gefitinib 250 mg/day at Kanagawa Cancer Center.

### Samples

Cytology specimens obtained by transbronchial abrasion were used for the analysis of EGFR gene mutations. All EGFR analyses were blinded with respect to the clinical response and demographic information before interpretation of the combined data.

### Isolation of DNA from specimens for cytologic diagnosis

Glass slides with cells that had been prepared for cytologic diagnosis by Papanicolaou staining were dipped in xylene until the coverslips naturally peeled off, and the slides were then rehydrated through a series of ethanol dilutions and air-dried. A Pinpoint Slide DNA Isolation System (Zymo Research, Orange, CA, USA) was used to extract DNA from the cells in accordance with the manufacturer's instructions. Briefly, an appropriate amount of a viscous solution (Pinpoint Solution, supplied in the kit) was applied over the area of cancer cells on the slide and air-dried to a thin film, together with the underlying cells. The film was then lifted with a blade, transferred to a tube containing a solution of proteinase K and digested at 55°C for 4 h. The tube was further incubated at 98°C for 10 min to inactivate the enzyme and then immediately quenched on ice. After vigorous vortexing, the tube was centrifuged and part of the supernatant was directly subjected to the following polymerase chain reaction (PCR)-based analyses.

### Mutation analyses by LH-MSA

The LH-MSA, a modified heteroduplex technique, was used to analyse the EGFR gene mutations. Briefly, two genomic DNA fragments spanning the mutation hotspots in exons 19 and 21 were amplified by PCR with the primers e19F and e19R (for exon 19) or e21F and e21R (for exon 21) (Table 1). At the end of the PCR amplification cycle, a specific LH probe for the detection of exon 19 mutations (e19LH) or exon 21 mutations (e21LH) was added to the PCR reaction solution at 500 nM. The mixture was then subjected to an LH cycle consisting of denaturation at 94°C for 2 min, annealing of the LH probe at 55°C for 15 s and extension of the LH probe by PCR at 68°C for 4 min. After the LH cycle, the product was separated by electrophoresis in a preformed native 10% polyacrylamide gel (Atto Inc., Tokyo, Japan) in Tris-glycine buffer (37.5 mM Tris, 288 mM glycine). Next, the gels were stained with SYBR Green I (Cambrex Bio Science, Rockland, ME, USA) and the DNA fragments were detected with a laser scanning imager (STORM860; Amersham Biosciences, Piscataway, NJ, USA). The bands representing LH DNAs were then excised and crushed in a small quantity of water, before an aliquot of each extract was re-amplified by PCR. The PCR products were subcloned into the pCR4TOPO (Invitrogen, Carlsbad, CA, USA) plasmid vector, and the nucleotide sequences were confirmed. All PCR amplifications and elongation reactions with LH probes were performed with Accuprime *Taq* polymerase together with a primer-template hybridisation-enhancing reagent (Invitrogen).

### Statistical analysis

The *χ*^2^ test was used to identify differences in the gefitinib responses between wild-type and mutant EGFR genes. The influence of each factor on the response to gefitinib was examined by logistic regression analysis. The Kaplan–Meier method was used to estimate the probability of survival, and differences in survival were analysed by the log-rank and Wilcoxon tests. Differences at *P*<0.05 were considered significant. All analyses were performed using StatView or Fisher's software.

## RESULTS

**Figure 1:**
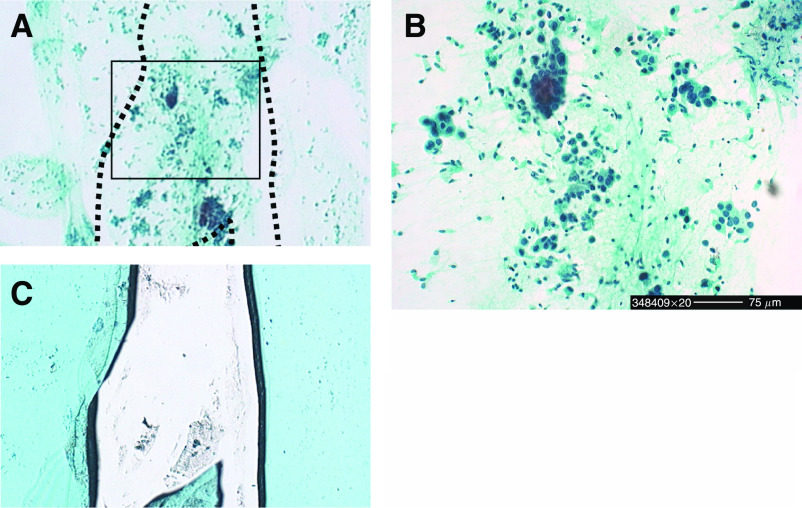
Removal of cytology specimens for analysis of the EGFR gene status. Glass slides previously prepared for cytologic diagnosis with Papanicolaou staining were dipped in xylene until the coverslips naturally peeled off, and the samples were then rehydrated for recovery of the cancer cells. (**A–C**) An area of cancer cells before (**A**) and after (**C**) removal by the PinPoint method is shown. In (**B**), the obtained cancer cells in the boxed area of (**A**) are shown at a higher magnification.

**Figure 2:**
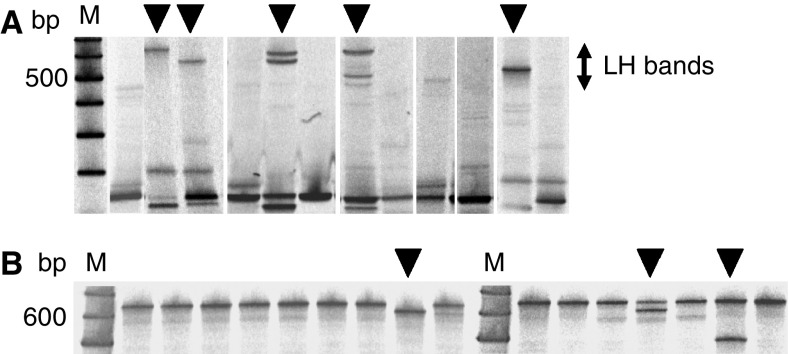
Detection of EGFR gene mutations from cytology specimens by LH-MSA. (**A**) Analysis of deletion-mutations in exon 19. Mutated products are identified as bands showing delayed mobility owing to heteroduplex formation in LH-MSA. The locations of the shifted bands are indicated by the vertical bar on the right. (**B**) Analysis of point mutations in exon 21. Mutated cases are indicated by arrowheads above the panels. A vertical arrow at the left side of (**A**) shows the area where LH bands appear. bp: base pairs, M: molecular size marker, LH: loop hybrid.

A feasibility study was carried out to determine whether cytology specimens obtained by transbronchial abrasion were applicable for analysis of the EGFR gene status. A total of 52 patients who were diagnosed cytologically as having class V NSCLC by transbronchial abrasion were entered into this study. A Pinpoint Slide DNA Isolation System was used to extract DNA from cancer cells alone on glass slides (Figure 1), and the EGFR gene status was analysed using LH-MSA as described in the Patients and methods. Analysis of the EGFR gene status using a few cancer cells was not possible in just two patients (3.8%), owing to insufficient amounts of the recovered DNA, but was possible in the remaining 50 patients (96.2%). Representative EGFR gene statuses of cytology specimens are shown in Figure 2. Deletion mutations in exon 19 were identified as bands showing delayed mobility owing to heteroduplex formation (Figure 2A), whereas point mutations in exon 21 were observed as extra bands (Figure 2B). (Table 2)

**Figure 3:**
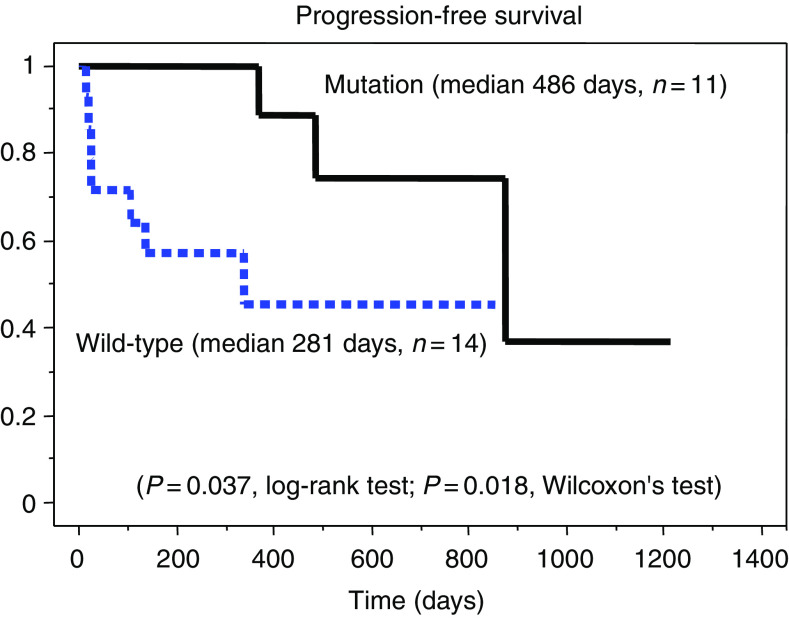
Progression-free survival curves according to the EGFR gene status, constructed using the Kaplan–Meier method. Patients with EGFR mutations have significantly longer progression-free survival than patients with the wild-type EGFR gene.

**Figure 4:**
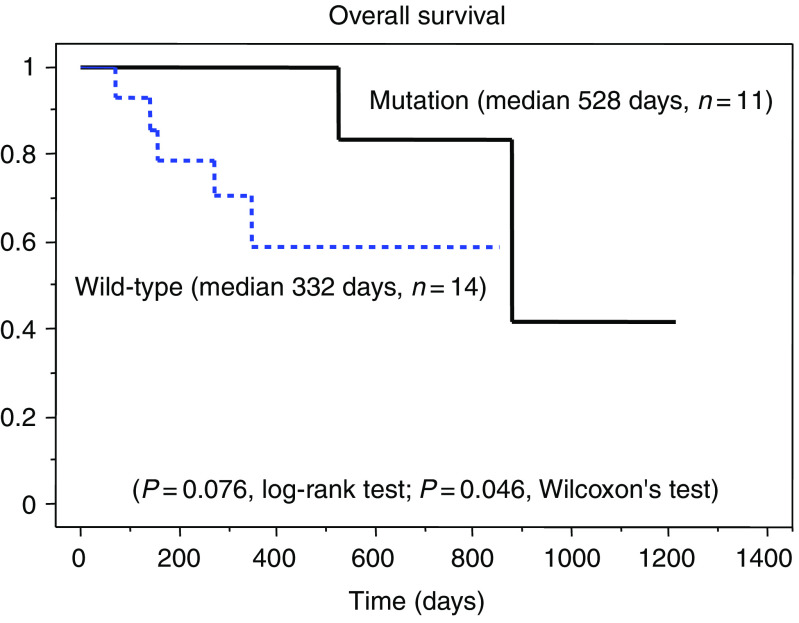
Overall survival curves according to the EGFR gene status, constructed using the Kaplan–Meier method. Patients with EGFR mutations show marginally, but significantly, longer overall survival than patients with the wild-type EGFR gene.

Among the 50 patients, 25 patients with metastatic lesions received 250 mg/day gefitinib treatment at Kanagawa Cancer Center (Table 3). Among them, 22 patients had been treated with one or two regimens of chemotherapy before starting gefitinib, but none received any further chemotherapy after the gefitinib treatment. Overall, eight of these patients were male and 17 were female, and they included 11 smokers and 14 non-smokers. Regarding the cancer types, 22 patients had adenocarcinoma, one had squamous cell carcinoma, one had non-small-cell carcinoma and one had undifferentiated carcinoma. We further divided the patients into an EGFR mutation group (*n*=11) and a wild-type EGFR group (*n*=14), and compared their EGFR statuses and clinical outcomes. Among the 11 patients with EGFR mutations, 10 showed tumour regression after gefitinib treatment and one showed no cancer progression over 1 year. Only two of 14 patients with the wild-type EGFR gene showed gefitinib-induced tumour regression. The response rate of patients with EGFR mutations was significantly higher than that of patients with the wild-type EGFR gene (90.9 *vs* 14.3%, *P*=0.00014, *χ*^2^ test). Logistic regression analysis revealed that EGFR mutations were the only significant factor contributing to gefitinib sensitivity (*P*=0.0016; Table 4). Patients with EGFR mutations showed significantly longer progression-free survival than patients with the wild-type EGFR gene (*P*=0.037, log-rank test; *P*=0.018, Wilcoxon test; Figure 3). Patients with EGFR mutations also showed marginally, but significantly, longer overall survival than patients with the wild-type EGFR gene (*P*=0.076, log-rank test; *P*=0.046, Wilcoxon test; Figure 4).

## DISCUSSION

We analysed cytological specimens from a total of 52 patients with class V NSCLC and were able to identify the EGFR gene status in 50 patients (96.2%). This is a very high percentage compared with previous studies in which the EGFR gene status was clarified in about 30% of patients using biopsy or resected tumour specimens (Tsao *et al*, 2005; Niho *et al*, 2006). Furthermore, the EGFR gene status identified using LH-MSA in the present study was well correlated with the antitumour effect of gefitinib. Responsiveness to gefitinib has been demonstrated in distinct subgroups of patients, such as women, patients who have never smoked, patients with adenocarcinoma and Asians (Kris *et al*, 2003; Miller *et al*, 2004; Thatcher *et al*, 2005). We carried out logistic regression analysis of various factors, and found that only EGFR mutations in cytology specimens represented an independent predictor for sensitivity to gefitinib. Taken together, these findings indicate that clarification of the EGFR gene status should be feasible in the majority of patients using LH-MSA, thereby making it possible to decide which patients would benefit from gefitinib treatment. Clinical experience has demonstrated that a patient with poor performance owing to respiratory failure caused by lymphangitis carcinomatosa responded to gefitinib treatment and showed an improved status with relief of dyspnoea (Patient No. 9 in Table 3). In general, such patients have invariably shown no response to anticancer drugs and experienced severe toxicities, thus contraindicating them for chemotherapy. Therefore, it would be clinically beneficial to examine the sensitivity of such patients to gefitinib before treatment.

Gefitinib is not currently a first-line anticancer drug, and is usually used after previous treatments with several conventional chemotherapeutic reagents. It is probable that the preceding chemotherapy may modify the sensitivity to gefitinib, as acquired cross-resistance of cancer cells to multiple anticancer drugs is a commonly encountered clinical phenomenon. Therefore, we consider that it is critical to evaluate the efficacy of anticancer drugs, including gefitinib, just before their use. The LH-MSA used in the present study requires only a small number of cancer cells, which may be sampled using common clinical procedures, such as collection of sputum, pleural effusion or peripheral blood. Our present findings suggest that the majority of patients could be tested in this manner for the presence of EGFR mutations, thus allowing selection of patients who would be likely to benefit from gefitinib treatment.

Our results confirmed that specific missense and deletion mutations in the tyrosine kinase domain of the EGFR gene are associated with the response to gefitinib. However, some of our patients without EGFR mutations also responded to gefitinib, suggesting that the clinical benefits of the drug cannot be explained only by the presence of EGFR mutations. Previous studies have demonstrated that the EGFR gene copy number is significantly associated with the response to gefitinib, and that gefitinib-treated patients showing EGFR gene amplification or high polysomy have significantly better responses, a longer time to progression and longer survival than patients with no or low EGFR genomic gain (Cappuzzo *et al*, 2005; Takano *et al*, 2005). Another study demonstrated an association between EGFR mutations and increased EGFR gene copy numbers in the human lung cancer cell line H3255 (Andrechek *et al*, 2000), although a large-scale study found that the presence of mutations was not correlated with either the expression or copy number of EGFR (Tsao *et al*, 2005). Therefore, determination of not only mutations but also the number of copies of EGFR is controversial for more certain clarification of likely responders to gefitinib. We are now planning a prospective study to examine whether the EGFR gene status revealed by cytology specimens using LH-MSA is able to select likely responders to gefitinib and long-term survivors.

## Figures and Tables

**Table 1:** PCR primers and LH-G probes for LH-MSA

e19F	ggactctggatcccagaaggtg
e19R	catttaggatgtggagatgagc
e21F	ggcatgaactacttggaggac
e21R	cttactttgcctccttctgcatg
e19LP	ggactctggatcccagaaggtgagaaagttaaaattcccgtcgctatcaaggaa
	ttaagagagcaacatctccgaaagccaacaaggaaatcctcgat
e21LP	cttactttgcctccttctgcatggtattctttctcttccgcacccagcag ^*******^
	agcccaaaatctgtgatcttgacatgctgcg
	or simply
	cttactttgcctccttctgcatggtattctttctcttccgcacccagcagagcccaaaa
	tctgtgatcttgacatgctgcg

LH-MSA=loop-hybrid mobility shift assay; PCR=polymerase chain reaction.

^*^Deleted nucleotides from the normal sequence.

The mutational hot spot is underlined.

**Table 2:** Type of in-frame deletion mutations in exon 19

G1 del(9)	del(L747-E749),A750P
G2 del(15)	del(E746-A750), c2481G>A
G3 del(15)	del(E746-A750)
G4 del(15)	del(R748-T751),L747S
G5 del(18)	del(L747-S752),E746V
G6 del(18)	del(L747-S752)

**Table 3:** Patient characteristics, EGFR gene status and clinical outcome

			**Diagnosis**						**Pretreatment**			**Gefitinib treatment**						
							**EGFR**											
**Patient**	**Gender**	**Smoke**	**Age (years)**	**PS**	**Stage**	**Cytology**	**Exon 19**	**Exon 21**	**1st**	**2nd**	**3rd**	**Age**	**PS**	**Effect**	**Ongoing**	**Progression- free survival after gefitinib (days)**	**Overall survival after gefitinib (days)**	**Alive**
1	M	Smoker	57	1	IV	non-sm	del(15)G4	W	NP+CPT+TRT	DP+TXT	—	58	1	PR	+	219	219	+
2	F	Non	63	1	IV	ad	del(15)G3	F856L	DP+Gem	—	—	66	1	PR	+	1214	1214	+
3	F	Non	43	2	IV	ad	del(18) G5	W	Tx+CPT	—	—	46	2	PR	−	876	878	−
4	F	Non	61	3	IV	ad	del(15)G3	W	Tx+CPT	—	—	64	1	PR	−	486	528	−
5	F	Smoker	60	1	IV	ad	W	L858R	Tx+CPT	—	—	61	1	PR	+	295	295	+
6	F	Non	56	0	IIA	ad	del(15) G2,G4	L858A/L858W	surgery	WBI	—	59	1	NC	−	367	537	+
7	F	Non	62	1	IIIB	ad	del(15) G4	W	Tx+CPT	—	—	64	1	PR	+	629	629	+
8	F	Non	55	1	IV	ad	del(18) G5	W	Tx+CPT	—	—	56	1	PR	+	516	516	+
9	F	Non	58	2	IV	ad	del(15) G3	W	Tx+CPT	—	—	60	4	PR	+	482	482	+
10	F	Non	47	1	IV	ad	W	L858A/L858R	Tx+CPT	—	—	50	1	PR	+	395	429	+
11	F	Non	60	0	IIIA	ad	del(15)G2	nd	surgery	NP+CPT	—	64	1	PR	+	817	817	+
12	F	Smoker	52	1	IIIA	sq	W	W	NP+CPT	DP+TXT	—	54	1	NC	−	138	141	−
13	M	Smoker	69	3	IV	udca	W	W	—	—	—	70	3	NC	−	107	274	−
14	M	Smoker	69	2	IV	ad	W	W	Tx+CPT	—	—	70	1	NC	+	436	436	+
15	F	Non	66	1	IV	ad	W	W	Tx+CPT	—	—	68	1	PR	+	308	308	+
16	M	Smoker	66	0	IIIB	ad	W	W	surgery	NP+CPT	TXT	68	1	PD	−	21	326	+
17	M	Smoker	61	1	IV	ad	W	W	Tx+CPT	—	—	62	1	PD	−	16	157	−
18	F	Non	65	1	IIIB	ad	W	W	Tx+CPT	—	—	67	1	NC	+	476	476	+
19	M	Smoker	70	1	IIIB	ad	W	W	NP+CPT	—	—	71	1	PR	+	254	254	+
20	M	Smoker	57	2	IV	ad	W	W	WBI	DP+Gem	—	59	1	NC	−	340	351	−
21	F	Non	69	1	IV	ad	W	W	Tx+CPT	—	—	71	1	NC	+	689	689	+
22	M	Smoker	41	1	IV	ad	W	W	DP+VNR	TXT+Gem	—	45	1	NC	+	852	852	+
23	F	Non	65	1	IIIA	ad	W	W	surgery	DP+VNR	—	72	1	PD	−	25	611	+
24	F	Smoker	80	1	IV	ad	W	W	NP+CPT	—	—	81	2	PD	−	23	69	−
25	F	Non	69	2	IV	ad	W	W	WBI	—	—	70	3	NC	+	338	338	+

ad=adenocarcinoma; CPT=irinotecan; DP=cisplatin; EFGR=epidermal growth factor receptor; F=female; G2=del(746E-750A); G3=del(746E-750A); G4=del(747L-751T) P741T; G5=del(747L-752S) E746V; Gem=gemcitabine; M=male; NC=no change; nd=not done; non-sm=non-small-cell carcinoma; NP=nedaplatin; PD, progressive disease; PR=partial response; PS=performance status; sq=squamous cell carcinoma; TRT=thoracic radiotherapy; Tx=paclitaxel; TXT=docetaxel; udca=undifferentiated carcinoma; VNR=vinorelbine; W=wild type; WBI, whole-brain irradiation.

**Table 4:** Logistic regression analysis of various factors that predict gefitinib effectiveness

**Variable**		**Odds ratio**	**95% CI**	***P-*value**
Gender	Female/male	0.233	0.036–1.513	*0.127*
Pathology	Ad/non-ad	0.5	0.039–6.353	*0.593*
Smoking status	Never/current	0.208	0.037–1.163	*0.074*
EGFR status	Mutation/wild	0.017	0.001–0.212	*0.002*

ad=adenocarcinoma; CI=confidence interval; EGFR=epidermal growth factor receptor.

Values in italics denote significance at *P*<0.05.

## References

[bib1] Andrechek ER, Hardy WR, Siegel PM, Rudnicki MA, Cardiff RD, Muller WJ (2000) Amplification of the neu/erbB-2 oncogene in a mouse model of mammary tumorigenesis. Proc Natl Acad Sci USA 97: 3444–3449 10716706 10.1073/pnas.050408497 PMC16259

[bib2] Cappuzzo F, Hirsch FR, Rossi E, Bartolini S, Ceresoli GL, Bemis L, Haney J, Witta S, Danenberg K, Domenichini I, Ludovini V, Magrini E, Gregorc V, Doglioni C, Sidoni A, Tonato M, Franklin WA, Crino L, Bunn Jr PA, Varella-Garcia M (2005) Epidermal growth factor receptor gene and protein and gefitinib sensitivity in non-small-cell lung cancer. J Natl Cancer Inst 97: 643–655 15870435 10.1093/jnci/dji112

[bib3] Fukuoka M, Yano S, Giaccone G, Tamura T, Nakagawa K, Douillard JY, Nishiwaki Y, Vansteenkiste J, Kudoh S, Rischin D, Eek R, Horai T, Noda K, Takata I, Smit E, Averbuch S, Macleod A, Feyereislova A, Dong RP, Baselga J (2003) Multi-institutional randomized phase II trial of gefitinib for previously treated patients with advanced non-small-cell lung cancer (The IDEAL 1 Trial). J Clin Oncol 21: 2237–2246 12748244 10.1200/JCO.2003.10.038

[bib4] Giaccone G, Herbst RS, Manegold C, Scagliotti G, Rosell R, Miller V, Natale RB, Schiller JH, Von Pawel J, Pluzanska A, Gatzemeier U, Grous J, Ochs JS, Averbuch SD, Wolf MK, Rennie P, Fandi A, Johnson DH (2004) Gefitinib in combination with gemcitabine and cisplatin in advanced non-small-cell lung cancer: a phase III trial – INTACT 1. J Clin Oncol 22: 777–784 14990632 10.1200/JCO.2004.08.001

[bib5] Herbst RS, Giaccone G, Schiller JH, Natale RB, Miller V, Manegold C, Scagliotti G, Rosell R, Oliff I, Reeves JA, Wolf MK, Krebs AD, Averbuch SD, Ochs JS, Grous J, Fandi A, Johnson DH (2004) Gefitinib in combination with paclitaxel and carboplatin in advanced non-small-cell lung cancer: a phase III trial – INTACT 2. J Clin Oncol 22: 785–794 14990633 10.1200/JCO.2004.07.215

[bib6] Kelly K, Gaspar LE, Chansky K, Albain KS, Crowley J, Gandara DR, Southwest Oncology Goup (2005) Low incidence of pneumonitis on SWOG 0023: a preliminary analysis of an ongoing phase III trial of concurrent chemoradiotherapy followed by consolidation decetaxel and gefitinib/placebo maintenance in patients with inoperable stage III non-small cell lung cancer. Proc Am Soc Clin Oncol 41: 637s

[bib7] Kris MG, Natale RB, Herbst RS, Lynch Jr TJ, Prager D, Belani CP, Schiller JH, Kelly K, Spiridonidis H, Sandler A, Albain KS, Cella D, Wolf MK, Averbuch SD, Ochs JJ, Kay AC (2003) Efficacy of gefitinib, an inhibitor of the epidermal growth factor receptor tyrosine kinase, in symptomatic patients with non-small cell lung cancer: a randomized trial. JAMA 290: 2149–2158 14570950 10.1001/jama.290.16.2149

[bib8] Lynch TJ, Bell DW, Sordella R, Gurubhagavatula S, Okimoto RA, Brannigan BW, Harris PL, Haserlat SM, Supko JG, Haluska FG, Louis DN, Christiani DC, Settleman J, Haber DA (2004) Activating mutations in the epidermal growth factor receptor underlying responsiveness of non-small-cell lung cancer to gefitinib. N Engl J Med 350: 2129–2139 15118073 10.1056/NEJMoa040938

[bib9] Matsukuma S, Yoshihara M, Kasai F, Kato A, Yoshida A, Akaike M, Kobayashi O, Nakayama N, Sakuma Y, Yoshida T, Kameda Y, Tsuchiya E, Miyagi Y (2006) Rapid detection of hotspot mutations of EGFR, BRAF and NRAS using the loop-hybrid mobility shift assay. J Mol Diagn (in press) 10.2353/jmoldx.2006.060030 PMC1867624 16931592

[bib10] Miller VA, Kris MG, Shah N, Patel J, Azzoli C, Gomez J, Krug LM, Pao W, Rizvi N, Pizzo B, Tyson L, Venkatraman E, Ben-Porat L, Memoli N, Zakowski M, Rusch V, Heelan RT (2004) Bronchioloalveolar pathologic subtype and smoking history predict sensitivity to gefitinib in advanced non-small-cell lung cancer. J Clin Oncol 22: 1103–1109 15020612 10.1200/JCO.2004.08.158

[bib11] Mitsudomi T, Kosaka T, Endoh H, Horio Y, Hida T, Mori S, Hatooka S, Shinoda M, Takahashi T, Yatabe Y (2005) Mutations of the epidermal growth factor receptor gene predict prolonged survival after gefitinib treatment in patients with non-small-cell lung cancer with postoperative recurrence. J Clin Oncol 23: 2513–2520 15738541 10.1200/JCO.2005.00.992

[bib12] Niho S, Kubota K, Goto K, Yoh K, Ohmatsu H, Kakinuma R, Saijo N, Nishiwaki Y (2006) First-line single agent treatment with gefitinib in patients with advanced non-small-cell lung cancer: a phase II study. J Clin Oncol 24: 64–69 16382114 10.1200/JCO.2005.02.5825

[bib13] Paez JG, Janne PA, Lee JC, Tracy S, Greulich H, Gabriel S, Herman P, Kaye FJ, Lindeman N, Boggon TJ, Naoki K, Sasaki H, Fujii Y, Eck MJ, Sellers WR, Johnson BE, Meyerson M (2004) EGFR mutations in lung cancer: correlation with clinical response to gefitinib therapy. Science 304: 1497–1500 15118125 10.1126/science.1099314

[bib14] Perez-Soler R, Chachoua A, Hammond LA, Rowinsky EK, Huberman M, Karp D, Rigas J, Clark GM, Santabarbara P, Bonomi P (2004) Determinants of tumor response and survival with erlotinib in patients with non-small-cell lung cancer. J Clin Oncol 22: 3238–3247 15310767 10.1200/JCO.2004.11.057

[bib15] Shepherd FA, Rodrigues Pereira J, Ciuleanu T, Tan EH, Hirsh V, Thongprasert S, Campos D, Maoleekoonpiroj S, Smylie M, Martins R, van Kooten M, Dediu M, Findlay B, Tu D, Johnston D, Bezjak A, Clark G, Santabarbara P, Seymour L, National Cancer Institute of Canada Clinical Trials Group (2005) Erlotinib in previously treated non-small-cell lung cancer. N Engl J Med 353: 123–132 16014882 10.1056/NEJMoa050753

[bib16] Takano T, Ohe Y, Sakamoto H, Tsuta K, Matsuno Y, Tateishi U, Yamamoto S, Nokihara H, Yamamoto N, Sekine I, Kunitoh H, Shibata T, Sakiyama T, Yoshida T, Tamura T (2005) Epidermal growth factor receptor gene mutations and increased copy numbers predict gefitinib sensitivity in patients with recurrent non-small-cell lung cancer. J Clin Oncol 23: 6829–6837 15998907 10.1200/JCO.2005.01.0793

[bib17] Thatcher N, Chang A, Parikh P, Rodrigues Pereira J, Ciuleanu T, von Pawel J, Thongprasert S, Tan EH, Pemberton K, Archer V, Carroll K (2005) Gefitinib plus best supportive care in previously treated patients with refractory advanced non-small-cell lung cancer: results from a randomised, placebo-controlled, multicentre study (Iressa Survival Evaluation in Lung Cancer). Lancet 366: 1527–1537 16257339 10.1016/S0140-6736(05)67625-8

[bib18] Tsao MS, Sakurada A, Cutz JC, Zhu CQ, Kamel-Reid S, Squire J, Lorimer I, Zhang T, Liu N, Daneshmand M, Marrano P, da Cunha Santos G, Lagarde A, Richardson F, Seymour L, Whitehead M, Ding K, Pater J, Shepherd FA (2005) Erlotinib in lung cancer-molecular and clinical predictors of outcome. N Engl J Med 353: 133–144 16014883 10.1056/NEJMoa050736

